# Infectious Foci, Comorbidities and Its Influence on the Outcomes of Septic Critically Ill Patients

**DOI:** 10.3390/microorganisms12081705

**Published:** 2024-08-18

**Authors:** Ana Maria Oliveira, André Oliveira, Raquel Vidal, João Gonçalves-Pereira

**Affiliations:** 1 Unidade Cuidados Intensivos, Unidade Local de Saúde Estuário do Tejo, 2600-009 Vila Franca de Xira, Portugal; ana.l.oliveira@ulsetejo.min-saude.pt (A.M.O.); andre.caet@gmail.com (A.O.); 2 Faculdade de Medicina, Universidade de Lisboa, 1649-028 Lisboa, Portugal; andreia.raquel@campus.ul.pt; 3 Grupo de Investigação e Desenvolvimento em Infeção e Sépsis, Clínica Universitária de Medicina Intensiva, Faculdade de Medicina, Universidade de Lisboa, 1649-028 Lisboa, Portugal

**Keywords:** infection foci, bacteremia, hospital-acquired infections, septic shock, ICU, SAPS II

## Abstract

Sepsis is among the most frequent diagnoses on admission to the intensive care unit (ICU). A systemic inflammatory response, activated by uncontrolled infection, fosters hypoperfusion and multiorgan failure and often leads to septic shock and mortality. These infections arise from a specific anatomic source, and how the infection foci influence the outcomes is unknown. All patients admitted to the ICU of Hospital de Vila Franca de Xira, between 1 January 2017 and 31 June 2023, were screened for sepsis and categorized according to their infection foci. During the study period, 1296 patients (32.2%) had sepsis on admission. Their mean age was 67.5 ± 15.3 and 58.1% were male; 73.0% had community-acquired infections. The lung was the main focus of infection. Septic shock was present in 37.9% of the patients and was associated with hospital mortality. Severe imbalances were noted in its incidence, and there was lower mortality in lung infections. The hospital-acquired infections had a slightly higher mortality but, after adjustment, this difference was non-significant. Patients with secondary bacteremia had a worse prognosis (one-year adjusted hazard ratio of 1.36, 95% confidence interval 1.06–1.74, *p* = 0.015), especially those with an isolated non-fermenting Gram-negative infection. Lung, skin, and skin structure infections and peritonitis had a worse prognosis, whilst urinary, biliary tract, and other intra-abdominal infections had a better one-year outcome.

## 1. Introduction

Sepsis, defined as a dysregulated host immune system response to infection, is a serious medical condition that can induce multiple organ dysfunction and death, and is one major cause of admission in intensive care units (ICU) worldwide [1,2]. Beyond the underlying inflammatory response, it can further promote immunoparalysis, which accounts for a worsening prognosis [3]. Furthermore, sepsis and, especially, septic shock can become refractory to therapy, with persisting hypotension and hypoperfusion. Therapeutics for septic shock must be quickly instituted [4], as these clinical conditions can be life-threatening, increasing the need for organ support measures, with a mortality rate as high as 40% across different studies [5].

Several epidemiologic studies have tried to characterize this phenomenon, particularly focusing on patients admitted to the ICU. However, suboptimal data were found due to the heterogeneity of patients’ characteristics and history, comorbidities, and infection sites, as well as the stage of the disease, together with a confusing nomenclature describing this syndrome. Systemic inflammatory response, activated by uncontrolled infection often arising from a specific anatomic source, has been identified as the major factor contributing to mortality in septic shock. Consequently, in most studies, researchers’ attention is mainly focused on sepsis, and the infection and its foci are less explored [6]. Once triggered, this over-exuberant systemic process was believed to progress independent of the inciting infection and, thus, patients with different types of infection have been treated equally [7].

Infections are normally classified according to their medical vs. surgical focus, and the place of acquisition, either the community or the hospital, due to the different associated microbiologies. Each of these sub-groups may be associated with different inflammatory responses and different microorganisms, and diverse therapeutic responses are to be expected. When clustering patients into similar characteristics and infection sites, specifically when patients were stratified by infection site, an important phenotypic variation in illness severity and mortality was found [8]. Nevertheless, the impact of the different infections on the clinical presentation and the adequate patient approach (including organ support therapy) is largely unknown. The anatomical origin of an infection can influence patient progression and clinical outcome, with an impact on mortality being attributable, for example, to some infection foci that facilitate rapid dissemination with an early hemodynamic disturbance [9]. Previous studies have shown that non-surgical patients have a significantly higher sepsis disease severity when compared to surgical patients, often presenting with severe underlying medical conditions or age-related immunosenescence that can exacerbate the severity of their septic condition and translate into a higher organ-specific Sequential Organ Failure Assessment (SOFA) score and mortality [10,11]. Regardless, in surgical patients, some specific mechanisms of infections can be present, as surgical interventions generate weak spots and surgical devices may be placed, contributing to the complexity of perioperative septic patients. Moreover, the impact of associated bacteriemia on clinical outcomes remains controversial [12], although they may be related to the influence of the infection site.

Recognizing the early signs of sepsis and septic shock, and their systematic approach, is crucial for timely interventions and better clinical results. The Surviving Sepsis Campaign [13] aims to increase awareness of sepsis among clinicians, develop more precise concepts, and create evidence-based guidelines for the management of sepsis. These guidelines were developed to maximize the speed and accuracy of the identification and management of sepsis, even when it is lacking the unique features of the underlying infection [8]. Furthermore, with the rapid implementation of evidence-based care, the epidemiology of sepsis has evolved, with a decrease in early mortality and progression to chronic critical illness, although its long-term outcomes are still largely unknown [7,14].

Moreover, understanding clinical differences based on infection foci may contribute to a better understanding of variation in the host response, and help guide adequate stewardship measures, including the allocation of resources and empirical antibiotic therapy. The personalization of patients’ therapy should be a priority for research as it maximizes the benefits and minimizes toxicity.

This study aimed to evaluate the characteristics of the different foci of infection in patients admitted to the ICU, including the characteristics of the host (comorbidities, age, clinical severity), the infection (sepsis vs. septic shock, the presence of secondary bacteremia), the resource consumption (organ support therapy), of the intensive care and hospital length of stay (LOS), and the mortality, both in the short-term (ICU and Hospital) and long-term (after 1-year of follow up). A comparison between hospital- and community-acquired infections was also performed.

## 2. Materials and Methods

### 2.1. Study Protocol

This was a retrospective, observational, cohort study. Clinical data were retrieved from the ICU electronic database, the hospital laboratory electronic system, and the Portuguese National Electronic Health platform.

All adult patients (>18 years old) consecutively admitted to the ICU of the Vila Franca de Xira Hospital, Portugal, between 1 January 2017 and 30 June 2023, were included. Patients were screened for infection complicated by sepsis or septic shock. Septic patients were segregated for further analysis. Patients infected with the SARS-CoV-2 virus were excluded.

Septic patients were divided according to their primary focus of infection and if the infection was acquired in the Hospital or the community. We considered infections diagnosed more than 48 h after the patient was admitted to the hospital to be hospital-acquired. To avoid dispersion, we classified infections according to the organ or system involved: lung, urinary tract, biliary tract, peritoneum, other intra-abdominal, endocarditis, skin and associated structures, and central nervous system. All remaining infections (including unknown focus) were classified as “other”.

Clinical and demographic data, including age, gender, and comorbidities, were collected. The ICU and hospital LOS were computed based on admission to the ICU and discharge time (from the ICU or the hospital, respectively). All patients were followed until hospital discharge or death, whichever occurred first. The ICU and hospital mortality rates were computed.

We searched the laboratory electronic database to retrieve all blood cultures collected within the interval between 48 h before and 48 h after ICU admission. Positive blood cultures (not deemed contamination) were identified. Patients were classified as having negative blood cultures only if these were collected and an infecting pathogen was not recovered.

Severity on clinical admission was assessed through Simplified Acute Physiologic Score (SAPS) II and the presence of septic shock. Standard mortality was computed as the ratio between hospital mortality and SAPS II-predicted mortality. The need for invasive organ support therapy was measured for all patients, including continuous infusion of vasopressors, renal replacement therapy (RRT), and invasive mechanical ventilation (IMV).

The records of patients discharged alive from the hospital were screened one year after ICU admission. Patients were classified as being dead or alive after one year of follow-up. The one-year patient all-causes mortality was calculated.

Death rates in the hospital and during the first year were calculated for each focus of infection, and grouped by the place of acquisition (community or hospital), the presence of secondary bacteremia, and the presence of septic shock.

The study protocol was approved by the Ethical Committee of the Vila Franca de Xira Hospital at the 26 January 2024 meeting (decision number 04/2024). Informed consent was waived due to the retrospective, observational nature of the study.

### 2.2. Statistical Analysis

Descriptive statistics were computed. Data were summarized as mean ± standard deviation or median (percentile 25 and 75), according to data distribution. Categorical variables were described as N (%). The chi-square test was used to compare categorical variables, whilst continuous variables were evaluated with Student’s *t*-test or the Mann–Whitney U test, according to data distribution. One-year survival curves were plotted, according to the focus of infection, the presence of bacteremia, and community- vs. hospital-acquired infections. The Wilcoxon test was applied. The Cox proportional hazards, adjusted for age, focus of infection, and SAPS II score, as appropriate, were computed.

We performed a logistic regression analysis, including positive blood cultures, place of acquisition, SAPS II score, septic shock, and comorbidities, to identify the adjusted odds ratio (OR), with a 95% confidence interval (CI) for hospital mortality, for each focus of infection. Also, the standard mortality ratio was calculated for each infection focus.

To address the impact of septic shock on mortality for each infection focus, we calculated the odds ratio for mortality, comparing patients with and without septic shock. We also computed the one-year survival curve for patients discharged alive from the hospital, with either sepsis or septic shock.

Statistical analysis was performed using IBM SPSS Statistics v.29.0 (IBM, Somers, NY, USA). All statistics were two-tailed, and the significance level was defined as *p* < 0.05.

## 3. Results

### 3.1. Demographics and Baseline Characteristics

During the study period, there were 4020 admissions to the Vila Franca de Xira Hospital ICU. Of these, 1296 patients (32.2%) presented sepsis on admission and were segregated for further analysis. Their mean age was 67.5 ± 15.3 and 58.1% were male. The lung was the most common focus of infection, accounting for 30.9% of the total, of which 73.0% were community-acquired. Some significant imbalances were noted (Table 1). Overall, 55% of the septic patients presented a community-acquired infection at ICU admission and 45% a hospital-acquired infection. The 183 patients infected with SARS-CoV-2 were excluded from further analysis.

The patients’ comorbidities are presented in Table 1. Arterial hypertension was diagnosed in 44.3% of patients and was the most common. Some imbalances were noted according to the infection focus which roughly parallel the age differences. Unsurprisingly, chronic obstructive pulmonary disease was more prevalent in lung infection, whilst chronic kidney disease was particularly common in patients with urinary tract infections (22.5% and 24.2%, respectively). Only 24.1% of the infected population did not present at least one of the studied comorbidities.

More than one-third of these patients were admitted to the ICU with septic shock. Patients with intra-abdominal or urinary tract infections had a higher prevalence of shock.

### 3.2. Community- and Hospital-Acquired Infections

On admission, 55% of the patients had a community-acquired infection. Pulmonary and urinary tract infections were more often community-acquired, whilst intra-abdominal infections commonly complicated patients already in the hospital. Curiously, 61.9% of endocarditis cases were classified as hospital-acquired.

No significant differences were noted in age or SAPS II on admission to the ICU. Moreover, the ICU LOS, the mortality, and the need for vasopressors or RRT were similar between patients with community- and hospital-acquired infections. However, this second group needed invasive mechanical ventilation more often and had a significantly higher in-hospital LOS and mortality after ICU discharge (Table 2).

Blood cultures were more often positive in hospital-acquired than in community-acquired infected patients, but this difference was not significant.

**Figure 1:**
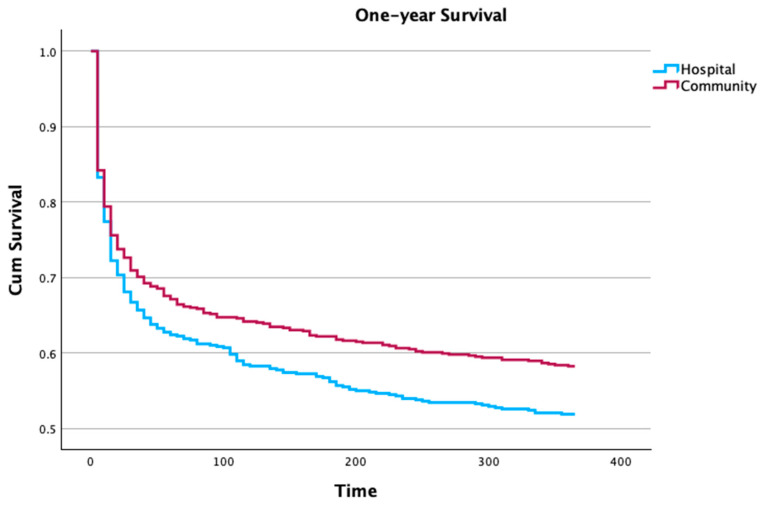
One-year survival curves according to the local of the infection acquisition (Wilcoxon statistic *p* = 0.046). However, the Cox proportional hazard ratio adjusted for SAPS II, age, and infection foci for 1-year mortality was 1.15, 95% confidence interval 0.96–1.36, *p* = 0.094.

One year after ICU admission, the mortality of patients with hospital-acquired infection was still significantly higher (Figure 1). However, after adjusting for the SAPS II, age, and foci of infection, this difference was no longer significant (Cox-proportional adjusted hazard ratio (aHR) 1.15; 95% CI 0.96–1.36; *p* = 0.094).

### 3.3. Positive Blood Cultures

In between 48 h before admission to the ICU and the first 48 h of stay, blood cultures were collected in 655 (50.5%) patients. Of Those, 24.4% returned positive, with slightly more positives in those with hospital-acquired infections (Table 2).

There was no significant difference in age between patients with bacteremia and those with negative blood cultures, although the SAPS II scores were significantly higher in the bacteremia group. Further, the need for organ support therapy was similar between the two groups. There were no significant differences in LOS, neither in the ICU nor in the hospital (Table 3).

Notwithstanding, the ICU mortality and hospital mortality were higher in the bacteremia group (Table 3).

**Figure 2:**
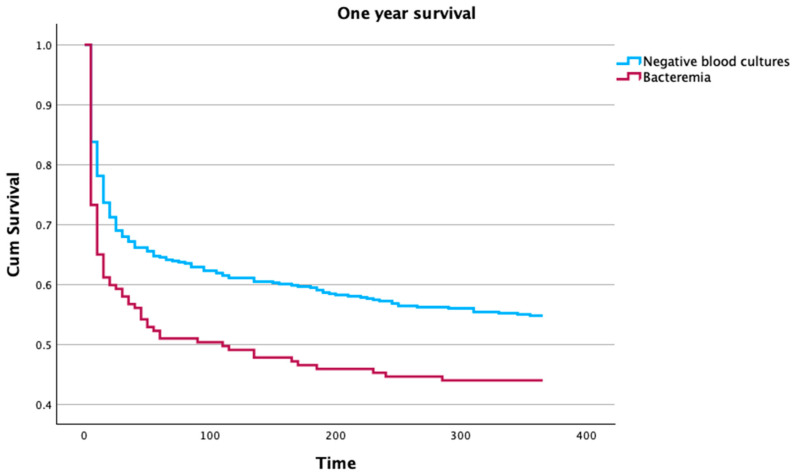
One-year survival curves for patients with bacteremia or negative blood cultures (Cox proportional age-adjusted hazard ratio 1.36; 95% confidence interval 1.06–1.74, *p* = 0.015).

This difference in mortality remained significant after one year of follow-up, even after adjusting for age (Cox proportional aHR 1.36, 95% CI 1.06–1.74, *p* = 0.015), Figure 2.

Some differences in outcomes were noted according to the different isolated bacteria (Table 4 and Table 5). Non-fermenting Gram-negative infected patients had a non-significantly higher hospital mortality (Chi-square test *p* = 0.402) and a significantly higher 1-year all-cause mortality (*p* = 0.022). Also, imbalances between bacteria, according to the different infection foci, were noted.

### 3.4. Clinical Outcomes

The hospital mortality of the population who were septic on admission to the ICU was 33.0%, including the 23.8% patients who die in the ICU. This was much higher than the mortality of the non-infected population (15.0%).

The hospital mortality was especially high in patients with endocarditis, CNS infections, and peritonitis (over 40%); on the contrary, urinary tract and biliary tract infections had the lowest hospital mortality rate. These differences could not be accounted for the difference in severity on admission, as the standard mortality ratio parallels this trend (Table 6).

Septic shock was associated with a significantly increased risk of dying in the hospital. This was more evident in patients with intra-abdominal infections (other than biliary tract infections) and lung infections. The hospital mortality rates of both endocarditis and CNS infections were not influenced by septic shock in our population, although those infections accounted for only 21 and 28 patients, respectively (Table 6).

Septic shock significantly increased the risk of mortality and resource consumption (Table 7). Although the ICU LOSs were similar (due to early deaths of patients with septic shock), the hospital total LOS (after ICU admission) was significantly longer. Of note, patients with septic shock were older, and more often had bacteremia and hospital-acquired infections (Table 7).

**Figure 3:**
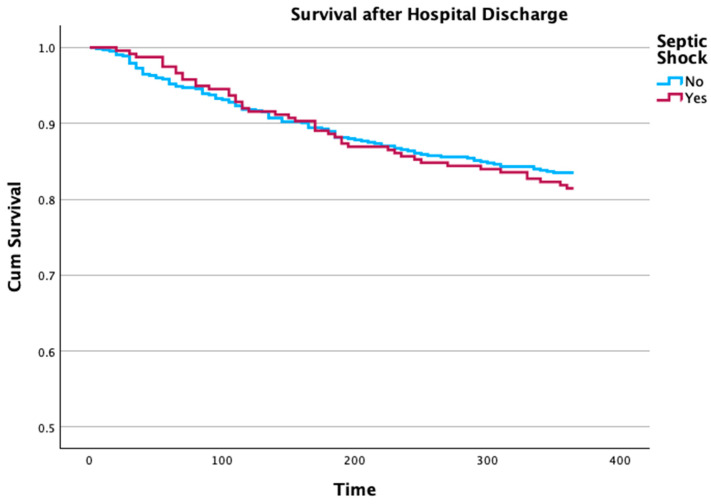
Survival curves of patients discharged alive from the hospital, after being admitted with sepsis or septic shock to the intensive care unit. There were no differences between the two populations (Wilcoxon statistics *p* = 0.583).

More than half of the patients admitted with septic shock died in the hospital, which was more than double the amount of patients with sepsis who died in the hospital. This difference remained throughout the first year of follow-up, but no differences were noted after hospital discharge (Figure 3).

In our hospital mortality model, urinary and biliary tract infections were again significantly associated with a better outcome. On the contrary, severity on admission (assessed by SAPS II), age, neoplasia, and chronic organ dysfunction (either kidney or hepatic) were all associated with higher mortality (Table 8).

Surprisingly, chronic hypertension was protective in patients who were septic on admission to the ICU.

**Figure 4:**
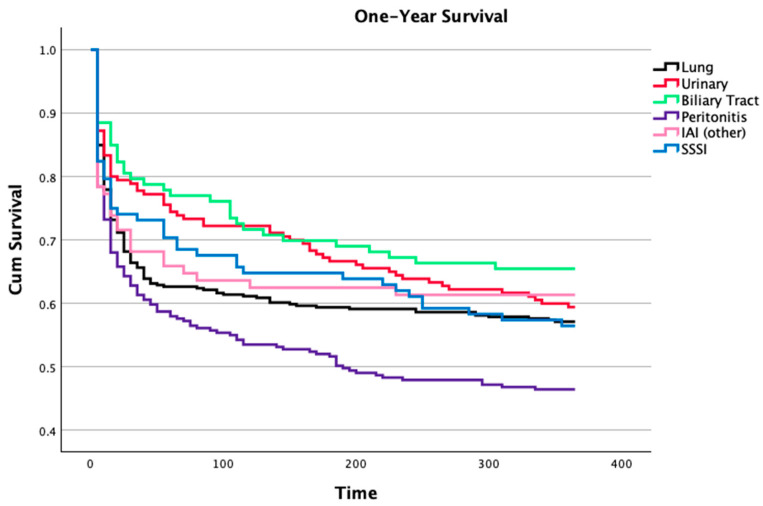
One-year survival of patients with sepsis after intensive care unit admission according to their infection focus. Patients with endocarditis, central nervous system infections, or non-classified were not represented (Wilcoxon statistics, *p* = 0.002). IAI—intra-abdominal infection, SSSI—skin and skin structures infection.

The one-year mortality was also influenced by the foci of infection (Figure 4). These differences parallel the hospital mortality, although a progressively worse prognosis of patients with peritonitis was noted (possibly related to the high rate of solid neoplasia, 30%). Lung and skin infections also presented a worse prognosis, whilst biliary and urinary tract infections and other intra-abdominal infections have a better one-year prognosis.

## 4. Discussion

We evaluated 1296 patients with sepsis on admission to the ICU (32.2% of total admissions), 37.9% of whom had septic shock. The most common site of infection was the lung (30.9%), followed by peritonitis (20.8%). Community-acquired infections accounted for 55% of infections, with significant imbalances, as there were more lung and urinary infections and less intra-abdominal infections or endocarditis. Septic shock was more frequent among patients with abdominal (54.5% IAI, 48.7% biliary tract, and 46.7% peritonitis) and urinary tract (47.3%) infections. Septic shock was associated with increased hospital mortality. This risk was higher for patients with peritonitis (OR 6.4, 95% CI 3.7–11.0), other IAI (OR 11.3, 95% CI 3.1–41.9), and lung infection (OR 6.8, 95% CI 3.9–11.8).

### 4.1. Infection Foci

Several potential mechanisms may influence the impact of the infection site on the clinical outcomes [7,15]. Some pathogens are more likely to cause infection at specific anatomical sites, and differences in virulence are to be expected [16,17]. Infected organs and systems may pose different risks for the patient, either from direct toxicity to the organ [18] or due to severe hypoperfusion and inflammatory dysfunction associated with sepsis [19]. In addition, anatomical differences may facilitate bacteria growth and differentiation [20].

Abdominal infections have been noted to be associated with a higher ICU mortality than other infection foci [21]. This mortality risk and the need for more organ support therapy [21] has been associated with a higher prevalence of septic shock or a significant drop in blood pressure upon surgery induction [22]. These worse outcomes may be linked to polymicrobial infection and its larger inoculum causing additional challenges in antibiotic therapy [23] and infection control. In addition, abdominal infections are more prone to being associated with septic shock [24]. Delays in surgical intervention and source control [25,26], as well as in the start of antibiotic therapy [27], are associated with a mortality increase. Failed source control is often difficult to identify and can be a cause of persistent infection [21]. In our study, intra-abdominal infections were also associated with higher rates of septic shock and bacteriemia. Biliary and urinary tract infections had a better outcome, possibly related to source-controllable infections. Consequently, quick identification of the correct site of infection is critical [28].

### 4.2. Community and Hospital-Acquired Infections

In our study, community-acquired infections accounted for 55% of the episodes of sepsis on admission to the ICU, less than previously described [29].

No significant differences were noted in patient severity on admission, although patients with hospital-acquired infections were slightly older (Table 2). A higher mortality rate has been described in hospital-acquired infections [30]. However, we only included patients with infection on ICU admission. Significantly, we did not find a difference in the 1-year mortality rate according to the place of infection acquisition, after adjusting for the site of infection, age, and severity. Nevertheless, the place of infection acquisition should influence the initial antibiotic therapy, alongside the local epidemiology and suspected source, previous antimicrobial exposure, and documented colonization with resistant bacteria [31].

### 4.3. Bacteremia

The impact of secondary bacteremia on mortality remains controversial, with conflicting results: although some studies reported no association of bacteremia with a worse outcome [32], others unveiled an increase in mortality, especially late mortality [12,33]. Healthcare-associated bloodstream infections are frequently due to multidrug-resistant strains [31], which are associated with a delay in the start of adequate antibiotic therapy. It is not clear if bacteremia increases disease severity by itself, or if it is only a marker of disease severity [12]. Age, illness severity, and immunosuppression were identified as risk factors for mortality [34].

In our study, we noted a low rate of positive blood cultures, only 24.4%, much lower than previously described [31,32]. However, we did not include ICU-acquired infections. Secondary bacteremia was associated with one-year mortality, even after adjusting for mortality (Figure 2), and this seems worse when a non-fermenting Gram-negative was isolated.

### 4.4. Septic Shock

The impact of septic shock on mortality is well known, although the heterogeneity of the syndrome leads to very different mortality rates, ranging from 15 to 56% with an average 30-day mortality of 34.7% [35]. Significant differences across continents were reported. With a more restrictive definition, Quenot et al. found a higher mortality [36], but the same was not reported in another prospective interventional study [37].

Abe et al. measured the impact of septic shock on the mortality rate according to the infection foci [32]. They noted a smaller effect on urinary tract and intra-abdominal infections than pneumonia. However, the authors did not differentiate between peritonitis and other intra-abdominal infections as we did in our study.

We were able to demonstrate the heterogeneity of the increased mortality risk associated with septic shock, as compared to sepsis. This may be related to a larger bacterial inoculum [38] and the lower efficacy of the antibiotic [39].

Sepsis is well known to impact the long-term prognosis of patients [40]. According to our data, this effect, a worse long-term outcome, seems to be related to sepsis itself, and not to clinical severity. Patients discharged alive from the hospital after sepsis had a similar follow-up mortality than patients discharged alive from the hospital after an episode of septic shock (Figure 3).

### 4.5. Mortality

Sepsis is largely known to affect the severity of organ dysfunction, the time in the ICU and the hospital, and the mortality [41]. This impacts its short- and long-term prognoses [40,42].

In our study, comorbidities including solid and haematological malignancies, as well as chronic renal and hepatic diseases, were independently associated with hospital mortality. Curiously, chronic hypertension proved to be protective. The risk factors for mortality seemed to be strongly influenced by the infection [43], but higher arterial pressure was always protective.

The foci of infection also influenced the mortality. In a prospective study, Storz et al. [7] showed significant heterogeneity in the time to resolution of immune response and organ dysfunction according to the site of infection, again showing a better prognosis of patients with urinary and skin-related infections, congruent to our findings.

Klastrup et al., in a retrospective study, identified 388 patients with sepsis or septic shock. After 90 days of follow-up, patients with a gastrointestinal infection had the highest mortality rate [44], although the small numbers preclude further conclusions.

In another study, non-surgical patients presented a significantly higher 90-day mortality (37%) compared to surgical sepsis patients (30%, *p* = 0.0457), including more organ dysfunction and more need for organ replacement therapies [11], highlighting potential differences in these populations.

In Figure 4 we present the differences in the long-term follow-up according to the infection foci. Lung and skin infections presented a worse prognosis, opposite to biliary and urinary tract infections and other intra-abdominal infections, which had a better long-term outcome.

### 4.6. Limitations

This study has several limitations, mainly related to its pragmatic design. It is retrospective and based only on clinical records. No specifically designed protocol was used to identify infections and their foci on ICU admission. Consequently, misclassifications might have occurred. Further, we relied mostly on clinical diagnosis and sub-classification may have occurred. In addition, it was single-center and generalization of our findings may be difficult. Although a large sample was used, some infection foci were under-represented. We included data from the time of the COVID-19 pandemic. Although we specifically excluded those patients, this may have changed the epidemiology of infection in the ICU. Notwithstanding, our study encompasses a six-and-a-half-year period, three times longer than the pandemic period. We did not collect data on the period before ICU admission, and some important information may have been missed.

Our study also has some strengths. It included a large cohort, which was followed for a long period. We evaluated all available blood cultures, including negatives. The SAPS II score was universally used in patients with an ICU LOS longer than 24 h.

## 5. Conclusions

Infection and sepsis are common in the ICU and are associated with a worse prognosis. The patients are relatively old and often have comorbidities. The infection foci strongly influenced the clinical severity, the need for invasive organ support therapy, and the short- and long-term mortality. Septic shock amplified the odds of death in the hospital but not during the follow-up period. Secondary bacteremia was associated with a worse prognosis.

## Figures and Tables

**Table 1:** Demographic and comorbidity characteristics of the population.

	Lung (N = 400)	Urinary (N = 182)	Biliary Tract Infection (N = 115)	Peritonitis (N = 270)	Other IAI (N = 88)	Endocarditis (N = 21)	SSSI (N = 108)	CNS (N = 28)	Other (N = 84)	Total (N = 1296)
Male sex	66.5%	50%	52.2%	58.1%	43.2%	61.9%	62%	50.0%	59.5%	58.1%
Age (years) *	66.3 ± 16.3	70.1 ± 11.8	71.3 ± 14.3	67.8 ± 15.4	67.0 ± 15.5	68.9 ± 15.1	66.6 ± 15.1	60.5 ± 15.6	65.0 ± 14.6	67.5 ± 15.3
Comorbidities										
Dementia	2.5%	2.2%	7.8%	1.1%	0.0%	0.0%	1.9%	0.0%	0.0%	28 (2.2%)
Diabetes	29.5%	30.2%	28.7%	26.3%	23.9%	42.9%	40.7%	39.3%	25.0%	383 (29.6%)
Arterial Hypertension	40.5%	47.8%	57.4%	44.4%	53.4%	47.6%	39.8%	42.9%	32.1%	574 (44.3%)
HF	18.3%	15.4%	9.6%	8.1%	13.6%	23.8%	22.2%	7.1%	10.7%	186 (14.4%)
Obesity	9%	14.8%	13.9%	13.3%	12.5%	4.8%	13.0%	7.1%	7.1%	149 (11.5%)
Cancer	9.3%	13.2%	11.3%	30%	13.6%	4.8%	5.6%	0.0%	9.5%	182 (14.0%)
Hematologic cancer	4.5%	3.3%	1.7%	3.0%	5.7%	4.8%	9.3%	3.6%	8.3%	58 (4.5%)
COPD	22.5%	13.2%	11.3%	9.3%	11.4%	14.3%	11.1%	3.6%	11.1%	189 (14.6%)
CKD	14.0%	24.2%	12.2%	13.0%	18.2%	33.3%	16.7%	7.1%	20.2%	209 (16.1%)
CHD	4.5%	2.2%	7.0%	7.8%	3.4%	0.0%	4.6%	0.0%	4.8%	63 (4.9%)
Community-acquired	73.0%	61.0%	35.7%	28.9%	55.7%	38.1%	51.9%	85.7%	64.3%	713 (55.0%)

Data presented as percentages, except *—mean ±square deviation. Chi-square test significant for Sex (*p* < 0.001), Dementia (*p* = 0.002), Arterial Hypertension (*p* = 0.01), HF (*p* = 0.002), Cancer (*p* < 0.001), COPD (*p* < 0.001), CKD (*p* = 0.007); Community-acquired infections (*p* < 0.001). IAI—intra-abdominal infection, SSSI—skin and skin structures infection; CNS—central nervous system; HF—heart failure; COPD—chronic obstructive pulmonary disease, CKD—chronic kidney disease, CHD—chronic hepatic disease.

**Table 2:** Differences between hospital- and community-acquired infections.

		Hospital	Community	*p*
Focus of Infection	Lung	108 (27%)	292 (73%)	<0.001
	Urinary	71 (39%)	111 (61%)	
	Biliary tract	74 (64.3%)	41 (35.7%)	
	Peritonitis	192 (71.1%)	78 (28.9%)	
	Other IAI	39 (44.3%)	49 (55.7%)	
	Endocarditis	13 (61.9%)	8 (38.1%)	
	SSSI	52 (48.1%)	56 (51.9%)	
	CNS	4 (14.3%)	24 (85.7%)	
	Other	30 (35.7%)	54 (64.3%)	
Bacteremia		28.0%	21.4%	0.055
Age		68.6 ± 15.4	66.6 ± 15.1	0.018 *
SAPS II		43.9 ± 19.2	44.3 ± 19.5	0.46 *
ICU LOS		5 [3–9]	5 [3–8]	0.99 **
Hospital LOS		14 [7–32]	11 [6–22.5]	<0.001 **
RRT		21.3%	23.1%	0.461
Vasopressors		56.3%	51.8%	0.117
IMV		41.7%	30.2%	<0.001
ICU Mortality		25.9%	22.2%	0.117
Hosp Mortality		36.4%	30.3%	0.024

Data presented as percentages, mean ± square deviation or median (percentile 25–75), according to data distribution. Chi-square test except * Student’s *t*-test and ** Mann–Whitney U test. IAI—intra-abdominal infection, SSSI—skin and skin structures infection; CNS—central nervous system; ICU = intensive care unit, LOS = length of stay, SAPS—simplified acute physiology score; RRT—renal replacement therapy; IMV—invasive mechanical ventilation.

**Table 3:** Prevalence of bacteremia and its impact on the outcomes.

		Bacteremia	Negative Blood Cultures	*p*
N		160	495	
RRT		28.1%	24.8%	0.407
IMV		32.5%	39.2%	0.134
Vasopressors		61.3%	56.4%	0.312
ICU LOS		5 [3–9]	5 [3–9]	0.46 **
Hospital LOS		13 [7–24]	11 [5–25.75]	0.47 **
ICU Mortality		37.5%	25.9%	0.006
Hospital Mortality		33.9%	45.0%	0.014
1-Year Mortality		56.1%	45.2%	0.022
Age		69.4 ± 13.6	68.0 ± 14.2	0.263 *
SAPS II		49.7 ± 19.9	45.6 ± 18.1	0.028 *
Focus of Infection				<0.001
	Lung	17 (7.6%)		
	Urinary	31 (34.8%)		
	Biliary tract	26 (44.8%)		
	Peritonitis	30 (27.0%)		
	Other IAI	12 (29.3%)		
	Endocarditis	6 (40.0%)		
	SSSI	16 (25.8%)		
	CNS	0 (0.0%)		
	Other	22 (47.8%)		

Data presented as percentages, mean ± square deviation or median (percentile 25–75), according to data distribution. Chi-square test except * Student’s *t*-test and ** Mann–Whitney U test. Community-acquired infections (*p* < 0.001); septic shock (*p* < 0.001). RRT—renal replacement therapy; IMV—invasive mechanical ventilation; ICU = intensive care unit, LOS = length of stay, SAPS—Simplified Acute Physiology Score; IAI—intra-abdominal infection, SSSI—skin and skin structures infection; CNS—central nervous system.

**Table 4:** Bacteria isolated in blood cultures and hospital mortality.

		N	Hospital Mortality
**Gram positive**		47	46.8%
	*Staphylococcus* spp.	14	46.7%
	*Streptococcus* spp	5	41.7%
	*Enterococcus* spp.	2	100%
	Other Gram Positive	1	33%
**Gram Negative**		99	41.4%
	*Klebsiella* spp	33	45.5%
	*Escherichia* spp.	42	35.7%
	*Proteus* spp.	2	50%
	*Citrobacter* spp.	6	50%
	*Enterobacter* spp.	6	66.7%
	*Serratia* spp.	6	50%
	Other Gram Negative	5	20%
**Non-fermenting Gram-negative**		14	60%
	*Pseudomonas* spp.	14	60%

**Table 5:** Differences in clinical outcomes according to bacteria isolated in blood cultures.

		Gram Positive	Gram Negative	Non Fermenting Gram Negative	*p*
N		47	99	14	
Age		67.9 ± 15.2	70.4 ± 13.4	68.1 ± 16.5	0.547 *
SAPS II		46.2 ± 20.2	51.13 ± 19.7	49.9 ± 21.8	0.473 *
Focus of Infection					
	Lung	70.6%	17.6%	11.8%	<0.001
	Urinary	6.5%	93.5%	0.0%	
	Biliary tract	3.8%	96.2%	0.0%	
	Peritonitis	13.3%	76.7%	10%	
	Other IAI	33.3%	41.7%	25%	
	Endocarditis	100%	0.0%	0.0%	
	SSSI	37.5%	31.3%	31.3%	
	Other	54.5%	36.4%	9.1%	
RRT		29.8%	29.6%	13.3%	0.408
IMV		31.9%	32.7%	33.3%	0.993
Vasopressors		55.3%	61.2%	80%	0.232
ICU LOS		6 [3–9]	5 [3–8]	4 [1–9]	0.24 **
Hospital LOS		16 [5–31]	11.5 [5–23.2]	8 [1–23]	0.47 **
ICU Mortality		36.2%	36.7%	46.7%	0.742
Hospital Mortality		46.8%	41.8%	60%	0.402
1-Year Mortality		31.8%	55.7%	71.4%	0.022

Data presented as N or percentage, mean ± square deviation or median (percentile 25–75), according to data distribution. Only the 160 patients with positive blood cultures were included. Chi-square test except for * Student’s *t*-test and ** Kruskal–Wallis and Mann–Whitney U test. Community-acquired infections (*p* < 0.001); septic shock (*p* < 0.001). RRT—renal replacement therapy; IMV—invasive mechanical ventilation; ICU = intensive care unit, LOS = length of stay, SAPS—Simplified Acute Physiology Score; IAI—intra-abdominal infection, SSSI—skin and skin structures infection.

**Table 6:** Hospital and one-year mortality accounting for foci of infection and septic shock.

	Hospital Mortality	Standard Mortality	One-Year Mortality	OR if Septic Shock*	95% CI
Lung	34.5%	0.88	42.8%	6.8	3.9–11.8
Urinary	22.5%	0.53	40.1%	4.8	2.2–10.7
Biliary	20.9%	0.46	33.9%	2.0	0.8–05.1
Peritonitis	40.4%	0.90	53.3%	6.4	3.7–11.0
Other IAI	31.8%	0.66	38.6%	11.3	3.1–41.9
Endocarditis	57.1%	1.49	66.7%	0.9	0.2–05.1
SSSI	29.6%	0.74	43.5%	4.4	1.8–10.6
CNS	42.9%	0.94	46.4%	1.4	0.2–11.7
Other	38.1%	0.92	47.6%	3.8	1.5–09.8

*—Odds ratio with 95% confidence interval for hospital mortality, if septic shock was present (compared with patients with sepsis, only). IAI—intra-abdominal infection, SSSI—skin and skin structures infection; CNS—central nervous system.

**Table 7:** Impact of septic shock on health resources and outcomes.

	Septic Shock	Sepsis	*p*
**RRT**	37.7%	12.9%	<0.001
**IMV**	52.1%	25.1%	<0.001
**ICU LOS**	5 [2–10]	5 [3–8]	0.741 **
**Hospital LOS**	11 [3–25]	7 [13–26]	<0.001 **
**ICU Mortality**	42.0%	12.8%	<0.001
**Hospital Mortality**	51.1%	22.0%	<0.001
**1-Year Mortality**	60.5%	35.0%	<0.001
**Age**	70.2 ± 14.1	65.9 ± 21.3	<0.001 *
**SAPS II**	51.8 ± 16.6	39.5 ± 16.6	<0.001 *
**Bacteremia**	35.6%	16.6%	<0.001
**Community-acquired infection**	34.1%	65.9%	0.002
**Hospital-acquired infection**	42.5%	57.5%	

Data presented as percentages, mean ± square deviation or median (percentile 25–75), according to data distribution. Chi-square test except * Student’s *t*-test and ** Mann–Whitney U test. RRT—renal replacement therapy; IMV—invasive mechanical ventilation; ICU = intensive care unit, LOS = length of stay, SAPS—simplified acute physiology score.

**Table 8:** Factors independently associated with hospital mortality.

	OR	95% CI	*p*
SAPS II	1.03	1.02–1.04	*p* < 0.001
Age	1.03	1.02–1.04	*p* < 0.001
Chronic hypertension	0.56	0.42–0.73	*p* < 0.001
Solid neoplasia	1.76	1.20–2.56	*p* = 0.004
Hematological neoplasia	1.83	1.00–3.33	*p* = 0.049
Chronic kidney disease	1.63	1.15–2.30	*p* = 0.006
Chronic hepatic disease	2.63	1.50–4.62	*p* < 0.001
Community-acquired infection	0.72	0.54–0.97	*p* = 0.029
Infection focus *			*p* < 0.001
Lung	0.93	0.54–1.60	*p* = 0.792
Urinary tract	0.41	0.22–0.76	*p* = 0.005
Biliary tract	0.33	0.16–0.67	*p* = 0.002
Peritonitis	0.80	0.45–1.43	*p* = 0.450
Other Intra-abdominal	0.63	0.31–1.31	*p* = 0.217
Endocarditis	2.64	0.90–7.72	*p* = 0.076
Skin and Skin structures	0.64	0.33–1.26	*p* = 0.201
Central nervous system	1.60	0.59–4.35	*p* = 0.359

Hosmer and Lemeshow 19.45 (*p *= 0.013). *—Other infection (control). OR—odds ratio; CI—confidence interval; SAPS—Simplified Acute Physiology Score.

## Data Availability

Dataset available on request from the authors.
